# Efficient and Safe Strategies for Intersection Management: A Review

**DOI:** 10.3390/s21093096

**Published:** 2021-04-29

**Authors:** Jian Wang, Xinyu Guo, Xinyu Yang

**Affiliations:** 1 College of Computer Science and Technology, Jilin University, Changchun 130012, China; wangjian591@jlu.edu.cn (J.W.); xinyug19@mails.jlu.edu.cn (X.G.); 2 Key Laboratory of Symbolic Computation and Knowledge Engineering of Ministry of Education, Jilin University, Changchun 130012, China

**Keywords:** intersection management, intelligent transportation system, congestion avoidance, green light optimized speed advisory, trajectory planning, intersection collision detection, abnormal vehicle warning

## Abstract

Intersection management is a sophisticated event in the intelligent transportation system due to a variety of behavior for traffic participants. This paper primarily overviews recent studies on the scenes of intersection, aiming at improving the efficiency or guaranteeing the safety when vehicles pass the crossing. These studies are respectively surveyed from the perspectives of efficiency and safety. Firstly, recent contributions to efficiency-oriented, intersection management overviews from four scenes, including congestion avoidance, green light optimized speed advisory (GLOSA), trajectory planning, and emergency vehicle priority preemption control. Furthermore, the studies on intersection collision detection and abnormal information warning are surveyed in the safety category. The corresponding algorithms for velocity and route management presented in the surveyed works are discussed.

## 1. Introduction

With the growth in urbanization and ownership of cars, the intersection has become one of the most central places on the road where the incidence of traffic accidents and congestion is increasing year by year. NHTSA’s National Center found [1] that in the 756,570 intersection-related crashes for driver-attributed critical reasons, the most frequent critical reasons were inadequate surveillance (44.1%), false assumption of another’s action (8.4%), turned with obstructed view (7.8%), illegal maneuver (6.8%), internal distraction (5.7%), and misjudgment of gap or other’s speed (5.5%). The investigation revealed that travel delays due to traffic congestion caused drivers to waste more than 3 billion gallons of fuel and kept passengers stuck in their cars for nearly 7 billion extra hours–42 h per rush-hour commuter [2]. Thus, it is necessary to ensure safety, improve traffic efficiency, save resources, and reduce pollution.

Currently there are two main vehicle-to-everything (V2X) technologies, whose primary application is focused on ITS, backed up by the key players of various automotive, telecommunication, and transport industries: Dedicated short-range communications (DSRC) and cellular vehicle-to-everything (C-V2X), respectively based on IEEE 802.11p and 3GPP LTE/5G NR. [3] The development of Intelligent Traffic System (ITS) and the appearance of Vehicular Adhoc Networks (VANETs), which refers to a set of smart vehicles used on the road [4], help vehicles communicate with others and hugely improve the efficiency of passing crossroads. The V2X network system implements information exchange through wireless communication technology. V2X includes four major categories: Vehicle-To-Vehicle (V2V), Vehicle-To-Infrastructure (V2I), Vehicle-To-Pedestrian (V2P), and Vehicle-To-Network (V2N). The collaboration of these four types is an important step to achieve autonomous driving and intelligent transportation. When applying V2X vehicle networking to ITS, the system needs to send status information (position, speed, direction, etc.) of the vehicle collected by radio frequency identification (RFID), sensors, etc. installed on the vehicle to surrounding vehicles accurately through vehicles and roadside units (RSU), vehicles, and vehicles along with other communication methods [5] in real time. After information is gathered, an effective message is extracted through data analysis and processing. Appropriate trajectories and speeds are calculated using corresponding algorithms, providing intelligent decision-making basis for vehicle running.

**Figure 1:**
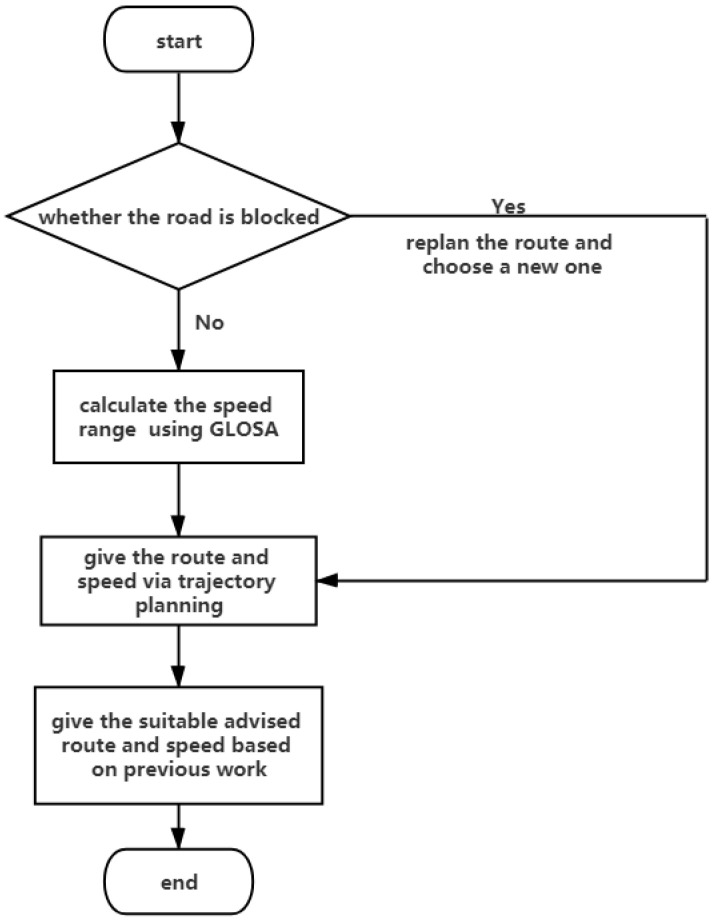
The process when closing to the intersection.

The scenes are divided into two board classifications: Safety and efficiency. The idea of this paper is shown in Figure 1. When approaching the intersection, the algorithms of collision detection and abnormal information warning start working to detect the hidden danger, guaranteeing cars safely passing the intersection. Two applications mentioned above are devoted to doing it. Sensors will detect whether the congestion exists and then the system uses algorithms to avoid it. After RSU sends the information about a traffic light, Green Light Optimized Speed Advisory (GLOSA) calculates the speed of passing the intersection without stopping. The metrics related to the speed advisory algorithms are given below. In order to find an applicable route, trajectory planning algorithms play an important role and give suitable routes. Combining methods of GLOSA and trajectory planning, we can obtain the route and speed of passing the intersection, which provides the driver with a better driving experience. To appreciate the particularity of emergency vehicles, the paper provides three methods on how to assist ambulances, police cars, and so on to cross an intersection promptly.

Based on these techniques, a new method is proposed to help a vehicle safely and quickly pass the intersection. We compare the main algorithms and choose relatively good methods in each scene. Vision-based detection is more cheap and has a wider range and its development is more mature, it is useful to use this this way to help us detect the barriers. Abnormal vehicle warning, control loss warning, and hazardous location warning are also finished with the assistance of a camera. VANET combined with image processing can be useful in good road conditions to avoid congestion. The part of GLOSA focuses on metrics for evaluating algorithms. In addition, LOSB-F is chosen to assist in passing the intersection without stopping. For trajectory planning, the Q-learning is selected for the route. The final part is a special circumstance. Both the traffic light and vehicles will receive messages to yield to it and help the emergency vehicles pass the intersection. Performance criteria from the China Academy of Information and Communications Technology is listed in Table 1. The main purposes of this paper are:

1. To reduce the number of acidents on the road;
2. To regulate the flow of vehicles and avoid congestion;
3. To eusure the transmission of information for safe driving and improve the efficiency of the vehicle pass rate.

The structure of this paper is as follows: Two ways of intersection collision detection and three cases of abnormal information warning are introduced in Section 2. Section 3 summarizes the scenes of efficiency which include four parts: (1) The methods of jam detection and congestion avoidance, (2) metrics and three main algorithms of GLOSA, (3) trajectory planning related to four different techniques, and (4) a particular situation: The emergency vehicle priority preemption control. This paper will select a better method from several algorithms based on each scenario and combine these methods to calculate a more accurate and appropriate speed and route. The last part concludes the paper and gives a further discussion according to the present works.

## 2. Safety

The method proposed in Section 2 and Section 3 allow vehicles to cross the intersection safely, efficiently, and with almost no pause. We need to continuously utilize intersection collision detection and abnormal information warning to ensure maximum safety during driving. The following briefly introduces several ways of improving efficiency and reduce road congestion.

### 2.1. Intersection Collision Detection

**Figure 2:**
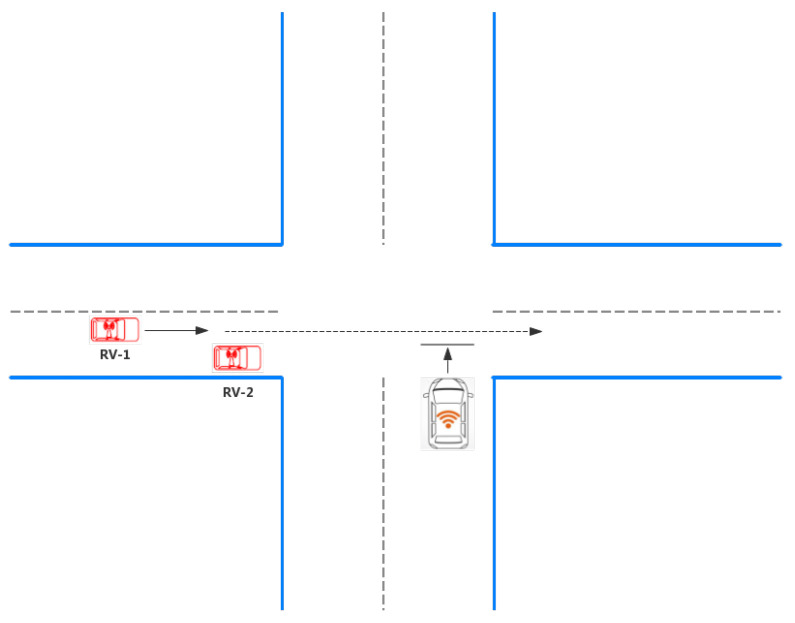
HV starts at the intersection.

**Figure 3:**
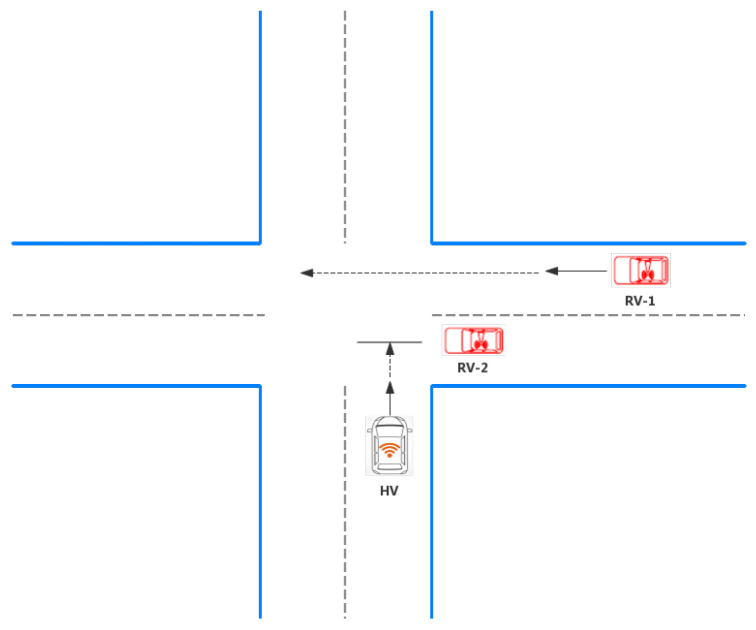
HV and RV drive to the intersection simultaneously.

In recent years, the number of deaths in road traffic accidents has continued to rise steadily, reaching 1.35 million in 2016 [6]. Lack of concentration, fatigue, and immature behavior are the leading causes of traffic accidents. Intersection Collision Warning is therefore introduced to alert the driver from an impending collision. There are two main scenarios for warnings at intersections: The HV starts at the intersection and the HV and RV drive to the intersection at the same time, which is shown in Figure 2 and Figure 3. Based on different input signals, the commonly used obstacle detection is divided into two types: Sensor-based and vision-based. The sensor-based dangerous target detection system has achieved excellent performance and has been widely used in autonomous driving for the strong environmental sensing capability of the sensors. However, laser radar sensors are too expensive to attain large-scale applications, and their ability to identify object categories is limited. Therefore, visual information is essential in an actual autonomous driving system. Inspired by human visual perception, the vision-based dangerous target detection system uses an in-vehicle camera to detect obstacles directly [7]. The following two detection techniques will be introduced separately and Table 2 compares these two ways.

#### 2.1.1. Sensor-Based Detection Technology

As mentioned previously in the congestion, sensors are primarily used to collect road information and we choose it to detect the situation of the road. The advances in sensors technologies enable real-time collection of high-fidelity spatiotemporal data on transportation networks. For example, a set of speed sensors that are spatially distributed along the street and can communicate through an externally determined network. The speed sensor observes the average speed value of the vehicle within a certain time interval, and uses a threshold-based method to generate a local prediction. Each sensor exchanges its local predictions with its neighbors and aggregates the local predictions it receives using a weighted majority aggregation rule to generate a final prediction [8]. However, this method makes assumptions about the transportation infrastructure that are currently unsupported, and do not explicitly model the detection delay and false alarm rate. Yasitha Warahena Liyanage et al. propose a two-stage approach. In the first stage, each sensor generates a decision using the Bayesian fastest change detection framework. In the second phase, individual sensor decisions are aggregated by an optimal stopping method, which optimizes the trade-off between aggregation costs and misclassification costs [9]. The proposed two-stage approach achieves up to 65.2% and 87% improvement in the average detection delay and probability of false alarm respectively, compared to the state-of-the-art. Liyanage [10] propose a Bayesian quickest change detection framework for accurate detection in near–real–time of freeway accidents based on speed sensors. To exploit the spatial correlation between sensors, and to improve the robustness of the proposed solution, four aggregation schemes are also proposed. We choose the second method to detect collision.

**Table 2:** Comparison of collison detection ways.

Detection Methods	Tool	Advantages	Disadvantages
Sensor-based detection [8,9]	radar, laser, lidar, etc.	Safe; measuring distance directly with less computing resources	Expensive, short range, less accurate
Vision-based detection [11,12]	camera	Cheap; wider range	More computations; affected by shadows from nearby buildings, overhead bridges or trees

#### 2.1.2. Vision-Detection Technology

Vision-based detection technology generally equips a camera in front of the car because the camera is cheaper than the sensor and can capture more traffic information, including object categories, object distances, traffic signs, and signals. Target detection obstacles are usually in the machine learning method, where the typical method usually consists of the proposed region, the features of the extracted proposed region, and the target recognition. An end-to-end target detection method [11] is proposed to improve the detection speed. It integrates the proposed region, feature extraction, and target recognition into a model to directly detect the target. The end-to-end approach can be used naturally for target detection in autonomous driving and is validated in actual autonomous driving data sets, such as KITTI. On this basis, a multi-task learning method based on a convolutional neural network (CNN) is given [12], which combines target detection with distance prediction. In order to facilitate distance prediction, the distance regression problem is transformed into a classification problem by discretizing continuous distance. According to the Cartesian product of object class and distance class, the joint optimization goal of the model is further proposed. Optimizing the target with a CNN is similar to a single-lens multi-box detector, taking into account both target detection and distance classification. The experiment proves mathematically that when the two tasks are not independent, the proposed Cartesian product-based combination strategy is better than the linear multi-task combination strategy in the traditional MTL. The system experiments show that the proposed method is superior to the existing SSD target detection method and the traditional MTL method. A traffic accident detection method, including a self-tuning iterative hard thresholding (ST-IHT) algorithm for learning sparse spatio-temporal features and a weighted extreme learning machine (W-ELM) for detection is proposed in this system [13]. Meanwhile, a two-point search strategy is proposed to adaptively find a candidate value of Lipschitz coefficients to improve the tuning precision.

### 2.2. Abnormal Information Warning(AIW)

**Figure 4:**
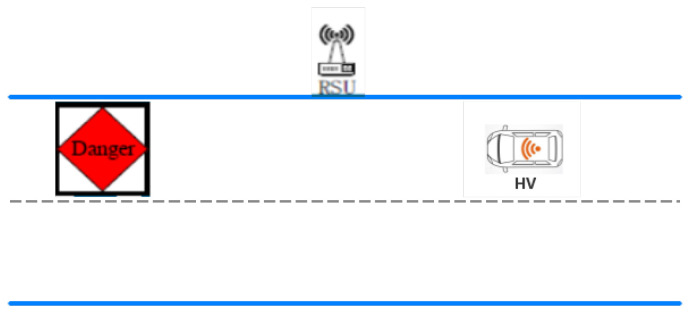
RSU reminds the HV of the danger.

As shown in Figure 4, when the driver is driving, they occasionally encounter abnormal information, such as faulty vehicles and bottomless pits. At this time, the host vehicle (HV) is needed to detect the road condition to tell the “driver”. The following describes three abnormal conditions.

#### 2.2.1. Abnormal Vehicle Warning (AVW)

When the remote vehicle (RV) turns on the fault warning light while driving, the HV recognizes that it belongs to the abnormal vehicle based on the content of the received message. The AVW function alerts the HV driver to the dangerous situation when the identified abnormal vehicle may affect the driving route of the vehicle. This application is applicable to abnormal vehicle reminders in the environment of urban and suburban ordinary roads, highway intersections, and entrances to loops and highways. Lei Song et al. [7] propose a new vehicle anomaly detection system based on in-vehicle monitoring system (IVMS). The video is taken by a camera mounted on the front of the car. The traffic information extracted by the system is combined with GPS and electronic maps to detect abnormal vehicles. The system can be installed on police cars, buses, and even private cars.

#### 2.2.2. Control Loss Warning (CLW)

When the RV is triggered by the brake anti-lock braking system (ABS), the body stability system (BSS), the traction control system (TCS), and the lane offset warning system (LOW) function, the main vehicle (HV) will alert the driver when the content of the received message identifies that the vehicle is out of control and may affect the HV driving route. Cuong Nguyen Khac et al. [14] propose a vehicle behavior analysis system for the Intelligent Driver Assistance System (IDAS). First, frame estimation is performed on all motion vectors of the moving target. Most safe moving vehicles are eliminated by using the new recommended threshold range and ROI settings. K-means clustering algorithm is used to cluster the remaining motion vectors to obtain segments of the abnormal candidate vehicles. The learned algorithm such as support vector machines (SVMs) and various features are used to classify the segmented candidate vehicles to eliminate non-vehicle candidate vehicles. The experimental results show that the method can detect the abnormally moving vehicles in front of the primary control vehicle during night driving. Table 3 lists three types of abnormal information warning.

#### 2.2.3. Hazardous Location Warning (HLW)

When the HV travels to potentially dangerous conditions (for example, there is deep water under the bridge, deep pits on the road, wet roads, sharp turns in front, etc.) and exists the risk of accidents, the HLW warns the HV driver. The European Union project NordicWay (an interchange server funded by the European Union CEF-program (connected to European equipment)), allows communication between vehicles from different manufacturers to be seamless with each other and the surrounding infrastructure and road operators have crossed the border to establish, implement, and have successfully tested it [15]. The project demonstrates the functionality of the communication channel by serving road engineering warnings (TMA-trucks, vehicle attenuators), hazardous location warnings (vehicles with hazard lights activated), and wet road warnings (vehicles with activated ESP or ABS). The project demonstrates this in the border areas of Sweden and Norway, as well as in the border areas of Sweden and Denmark.

### 2.3. Summary

This section mainly summarizes the collision detection tools currently in use, including sensors, visual target detection, and general sensor-based object detection. With the help of these hardware, it mainly talks about three abnormal information warning, including abnormal vehicle warning, out of control warning, and some dangerous conditions in the road warning, such as road sag.

## 3. Efficiency

### 3.1. Jam Detection and Congestion Avoidance

**Figure 5:**
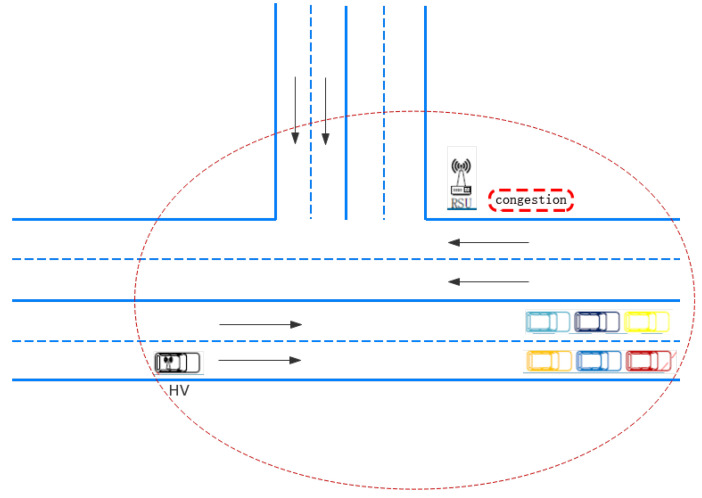
Typical scene of congestion ahead of the HV.

Road congestion has essential economic and social impacts in modern society, such as wasting gasoline, affecting efficiency, and annoying drivers [16]. With the increasing number of vehicles, road congestion has become increasingly serious, which is shown in Figure 5, thus the application of congestion reminders has become necessary in driving. This section focuses on road congestion detection techniques and how to help vehicles avoid congestion.

#### 3.1.1. Jam Detection

In any transportation hub system, using a unitary technology to detect whether the road is congested has considerable errors. At present, the mainstream detection technologies used to identify congestion are mainly divided into three categories: Image processing, sensing techniques, and probe-vehicles-based techniques.

The idea of image processing is to capture real-time images with a camera or video to calculate the density of cars so as to infer whether congestion will occur. Image processing chiefly consists of three methods: The first is background extraction, which provides complete information about the external features, but the problem is that this technique is particularly sensitive to changes due to illumination and external events. To solve this problem, Li et al. introduce an adaptive update of background extraction and propose an improved algorithm based on historical statistics combining the multi-frame average method [17]. In [18], the author developed a method to extract jam events by analyzing the road background feature using multiple background images. The extraction of background image is done by performing a difference on three consecutive frames. In the extracted background image the corner feature is analyzed to detect traffic jam. Results of the experiments show that the algorithm is robust and in real time. Besides, a scheme for assessing the state of congestion from the operation of cross-correlation between successive frames transmission in a CCTV (closed-circuit television) system is proposed [19]. The tests of this method are realized on a standardized dataset at different times of the day. Thus the results obtained make it possible to alert about traffic jams. Simple background image extraction is unstable in urban traffic videos, so the improved algorithm overcomes the image blur and the difficulty of tracking at the peak of traffic when utilizing the simple multi-frame average method. The Background Local Binary Pattern (BGLBP) handles a large number of illumination changes that occur during background extraction, and the algorithm is evaluated in three different ways [20]. The results shows that background extraction becomes more precise than before. A new method [21], which fits under the framework of mathematical morphology, is based on a recently developed textural descriptor termed as the Morphological Texture Contrast (MTC). In this work authors have employed the bright and dark top-hat transformations to handle the bright and dark features separately. Both bright and dark features extracted are subjected to the MTC operator for identifying the texture components, which in turn are used to enhance the textured parts of the original input image. Subsequently, this method is employed to segment the bright and dark textured regions separately from the two enhanced versions of the input image and the partial segmentation results obtained are combined to constitute the final segmentation result.

Nonetheless, the vision-based approach faces severe changes caused by illumination, shadows, swaying trees, and moving clouds. Therefore, the authors propose a real-time algorithm [22] that performs background and front-segment segmentation without using background modeling to overcome the above problems. In the first step, an improved block-based frame difference method is established to quickly detect moving targets without being affected by rapid illumination changes. In the next step, the dual foreground fusion method is used for accurate target region extraction. In the third step, a texture-based target segmentation method is used, which separates each car from the merged foreground image spots and removes the shadows. In the fourth step, a pseudo foreground filtering method based on the concept of motion entropy is proposed to remove the false target region caused by the rickety tree or the moving cloud. Finally, a texture-based target tracking method is used to track each detected target and then apply a virtual loop detector to calculate the traffic flow. However, when it comes to disasters and significant events, collecting data can be complicated whether it is background or foreground. Using aerial images can solve this problem. Therefore we use satellites to obtain high-resolution images and analyze traffic density based on photos to estimate whether congestion will occur. Ariel images enhancement techniques, such as interpolation algorithms, decimated wavelet with interpolation, wavelet zero paddings (WZP), and hybrid wavelet decomposition (HWD), can improve the calculation accuracy of traffic density [23].

On the highway, sensor technology is widely used because of the high traffic volume and fast speed, and the use of image processing technology to determine whether the blockage is rare. Sensors are classified as invasive and non-invasive. Non-invasive technologies are based on video image processing, optics, and ultrasound as well as lidar and microwave radar. The inductive loop, magnetometer, and pressure switch types are three main sorts of the current intrusive technologies. Inductive loop (IL) sensors installed at selected points of road infrastructure can be used for anonymous vehicle tracking in the application to efficient traffic control and calculates the passing vehicle at a certain point. However, various signal conditioning systems (SCS) cause disparities between the vehicles magnetic signature (VMS). An approach is proposed to increase compatibility between VMS from impedance SCS and generator-based SCS. A proper electrical model of a generator-based SCS is used to produce a compatible VMS from an impedance SCS [24]. In [25], the authors propose a method based on the single-chip microcomputer inductive loop vehicle sensor, which uses an automatic calibration algorithm based on the k-means clustering. The circuit is simple, and the signal processing does not require intensive computing power and can be implemented on a low-cost microcontroller. Inductive loops are a cost-effective solution, but when they are installed on poor road surfaces, the failure rate is high, resulting in reduced road life and obstructing traffic during maintenance and repair. Magnetic sensors are not affected by weather conditions and are suitable for large-scale deployment and time-sustainable detection for traffic information acquisition, thus providing more stable and reliable detection data than other technologies. The authors [26] introduce a multi-functional wireless magnetic sensor and propose an alternative, efficient and accurate detection method to solve the drift named “Wake effect” caused by the weather environment and the traffic flow environment. Another paper improves the performance of SCAN and Decision Algorithm (SDA) [27].The new algorithm, based on magnetic sensors, adds a two-pass moving average filter to improve the signal to noise ratio (SNR) after analog-to-digital conversion. The improved mathematical capabilities allow us to capture additional features of vehicle orientation and classification. The performance of the proposed algorithm is evaluated by field real-time experiments in a designated road section. The results show that the roadside magnetic sensors can improve vehicle detection, counting, travel time index, and classification in low speed and crowded traffic conditions. In addition, since acoustic sensors are cheaper than other types of vehicle sensors, and acoustic characteristics are robust to the environment changes like light, weather, etc., cross-microphone arrays are used [28] to collect roadside acoustic signals. The built-in lane detection module then automatically detects the lane position. Finally, based on the collected signals and the detected lanes, different measurement indexes reflecting road conditions and traffic quality are derived.

Probe-vehicle-based techniques mainly use two technologies: GPS-based and smart-phone-based technologies. The widespread use of car GPSs ensures that we can get traffic data and detect blocking information. Using low-cost GPS equipment to monitor traffic conditions in real-time, the paper [29] proposes a new real-time traffic evaluation coupling method, which can successfully match more than 98% of GPS data with service routes. The traffic network evaluation result shows that the method has reasonable practicability and can obtain more accurate vehicle information, so that we can timely use the algorithm to avoid congestion, thus alleviating traffic flow. With the development of smartphones, the use of GPS on smartphones has become more widespread. Using the collective intelligence recorded by smartphones, a sensor data platform is proposed for sensing, detecting, and visualizing road surface roughness and driving behavior [30]. The authors collected real driving records from different roads in Hyderabad and India, comparing the significant differences in visual conditions in terms of road conditions and driving behavior.

The sensor is selected as the detection tool in this paper, due to the low judgment accuracy of the current image processing technology and the fact that GPS loading is likely to reveal the user’s location privacy, thus causing malicious attack.

#### 3.1.2. Congestion Avoidance

With the increasing traffic congestion, the need for congestion avoidance technology in traffic management is growing. These technologies utilize various methods and strategies to avoid traffic congestion. These commonly used techniques include using a prediction algorithm, VANET, and signal timing optimization to predict and avoid congestion at common road network bottlenecks.

The driver can be informed by using prediction algorithm that the route is changed from a crowded area to a non-crowded area so that strategies such as vehicle diversion have also been proven to be an excellent choice to avoid congestion. Hu et al. [31] propose the actual urban traffic simulative model (AUTM) to predict and avoid traffic jams. The map and transfer (MT) transformation method in AUTM is used to obtain the actual urban cellular space and to optimize vehicle dynamics taking advantage of spatial evolution rules. The authors conduct a large number of experimental simulations in several real cities and the results show that the accuracy of traffic congestion prediction exceeds 89%. Nevertheless, how to apply the dynamic strategy of traffic lights to improve traffic flow is not considered. In the research [32], the authors propose an advanced dynamic traffic network prediction and navigation model. Compared with the traditional shortest path algorithm which considers the static network as the core, the first part of this guiding method considers the potential traffic jam, gives the optimal driving suggestion for different times of day, designs the equilibrium Markov chain model, and this method is used to dispatch vehicles to alleviate urban congestion. Inspired by the pooling operation in deep learning, a representation framework [33] for traffic congestion data in urban road traffic network is proposed. The framework consists of a grid-based urban road traffic network partitioning and a pooling operation that reduces multiple values to aggregate values. We also recommend using a pooling operation to calculate the maximum value in each grid (MAV). Raw snapshots of traffic congestion maps are converted and represented as a series of matrices that are used as inputs to the Spatio-temporal Congestion Prediction Network (STCN) to evaluate the validity of representation in predicting traffic congestion. M. A. Abdelwahab et al. [34] proposed a fast and reliable traffic congestion detection method based on deep residual learning and video dynamic modeling of motion trajectory. This method makes use of motion and deep texture features effectively, overcomes the limitations of existing methods, and uses an effective representation learning method to capture the underlying structure in traffic video by modeling the evolution of texture features. This representation provides a significant improvement in detection results under all weather conditions.

The basic idea of the prediction algorithm is to predict based on previous historical data, which is not friendly to the real-time nature of traffic. The full application of VANET solves this problem. A real-time queue length estimation method based on connected vehicle data is proposed [35]. The probe data is used to identify the stop state of the connected vehicles (CVs). On this basis, a queue length time series based on the stop time and the position of CVs is established to describe the queuing process of the intersection. Considering the statistical average traffic flow, the queue length time series and the stop state of arrival characteristics of the real-time CV in the current cycle, it is serviceable to use the linear fitting method to predict the critical queuing time and the Markov model to estimate the real-time queue length. Another paper studies queue length estimation for networked vehicles equipped with distance measurement sensors [36]. By extending the previous formula, a simple plug and play model is established to estimate queue length without requiring ground truth queue length. This method is simple to implement and can be used in the traffic signal circular queue with known phase length. The derived model was evaluated using data from microscopic traffic simulations.

Intelligent traffic light control can alleviate traffic congestion in an emergency and avoid future traffic congestion. Many algorithms have been optimized for signal timing to improve traffic flow. Vishu Gupta et al. propose an intelligent control method of traffic lights to manage congestion events by controlling the order in which the traffic lights turn green and modifying the time when the traffic lights turn green. In this paper, Hopfield Neural Network (HNN) is used to obtain the optimal traffic light sequence of a single intersection (node) and genetic algorithm is used to solve the optimal green time of traffic lights in the four-phase road network [37].

The summary of congestion avoidance is shown in Table 4. An algorithm based on VANET is chosen to check and predict the road congestion, because the real-time performance of the prediction algorithm is not adequate and the realization of intelligent traffic lights is difficult.

#### 3.1.3. Summary

In the blocking inspection, we mainly use image processing technology, sensor technology, and probe-vehicle-based techniques. Since image processing has high requirements for pictures and is easily affected by illumination and external events, it is only suitable for good road conditions. However on complex roads like highways, we prefer to use sensor technology. Relying on data from GPS and smartphones is also a good option when research budgets are limited.

### 3.2. Green Light Optimized Speed Advisory (GLOSA)

**Figure 6:**
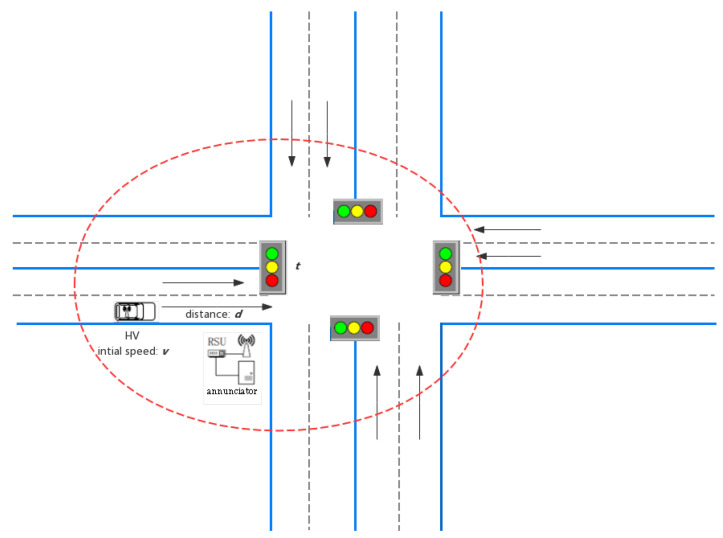
Green wave speed guide scene.

The GLOSA system has the effect of reducing CO${}_{2}$ emissions and fuel consumption, and avoiding unnecessary stops at intersections. GLOSA is divided into two situations: Signal light and no signal light. Since no signal light is close to platooning’s thought, only the signal light is discussed in this chapter, which is shown in Figure 6. When the vehicle approaches the intersection, the GLOSA system will give a reasonable recommended speed based on current and future traffic lights. These systems have been evaluated by a variety of simulations. This article will introduce the technical evaluation elements and environmental assessment elements of the system, as well as several mainstream GLOSA algorithms.

#### 3.2.1. Evaluation

Based on experience in actual testing and related work, Stahlmann et al. define a comprehensive set of newly created metrics for the technical evaluation of the GLOSA system, including latency, end-to-end delay, message delivery ratio, packet delivery ratio, stability of the prediction, and information distance. They cover all relevant system components required for GLOSA system functionality, including system and communication performance, as well as application-related measures and infrastructure aspects. Together, they allow for a comprehensive assessment and analysis of GLOSA performance [38].

The above criteria are technical standards proposed for algorithm optimization, but in actual simulation, we tend to look at more intuitive standards: ${\mathrm{CO}}_{2}$ emission, fuel consumption, and travel time. Even in high-density traffic conditions, the paper [39] points out that GLOSA can improve fuel economy and reduce carbon dioxide emissions by an average of about 10%. HOwever not every time GLOSA can work, the simulation results show that when the traffic per hour exceeds 400, GLOSA will lose the effect we want and be less environmentally friendly than conventional vehicles.

#### 3.2.2. Three Algorithms of GLOSA

With the assistance of VANET, the vehicle OBU is responsible for receiving information from the RSU and knowing the traffic light time plan of the intersection in advance, thereby determining the speed of the vehicle passing through the intersection under the condition of avoiding the red light according to different algorithms. The green wave speed is generally calculated based on the signal time and the distance from the signal light, so we will introduce the basic computational formulas in theory and some different ways in actual implementation.

As shown in Figure 6, *d* is the distance to the intersection, *v* is the initial speed, *t* is the time to reach the traffic light plus the remaining time for the next green phase ($t_{red}$ or $t_{yellow}$+$t_{red}$ respectively), and *a* is the acceleration, the basic rules of motion, given by (1)–(3) [40]:

(1) $$d=v*t+1/2*a*t^{2}.\;$$

The time to reach the traffic light ($t_{TL}$) can be calculated as shown in (2):

(2) $$t_{TL}=\left\{\begin{array}{cc} d/v, & a=0\\ -v/a+(v^{2}/a+2d/a), & a\neq 0. \end{array}\right.\;$$

The target speed ($v_{t}$) for the red light phase is calculated using (3):

(3) $$v_{t}=\left(2*d\right)/t-v.\;$$

Based on these equations, predecessors propose many different algorithms. A number of conventional vehicles currently in the sale are equipped with Adaptive Cruise Control (ACC) auxiliary systems, such as the Weilai ES8, the Ford Co-Pilot360TM, and the BMW 5 Series. The ACC equipment-connected vehicle travelling at the signalized intersection is often taken over by the driver without considering the signalized intersection constraints, and the vehicle speed changes obviously. In response to this problem, using the upcoming traffic signal information as the upper controller of the ACC system, Qi et al. [41] propose an ecological driving intelligent prediction model (IDM) based on V2X communication. For the intersection signal constraint problem considered in IDM, it is used as a virtual front vehicle before the red light, and there is no obstacle to deal with when the green light is on. In order to reduce the idling time at the intersection, a crossroad model method is proposed to predict the arrival time and the downstream queue discharge time. The article also proposes an eco-driving model combined with a deceleration strategy to resolve the combined signal phase and time (SPaT) and vehicle state constraints. Numerical simulations show that the speed profile generated by the eco-driven model is a speed consultant, which can reduce idling time and fuel consumption level near the signalized intersection. In order to ensure that CV followers can track the leader’s speed and interval time while achieving uniform performance, a conformance-based CV queue model [42] considering V2X network topology is proposed. Two crossing decision methods considering the duration of yellow light were proposed to determine the average expected speed of the leader and prevent the leader from running the red light. The safe and energy-saving oriented SAM based on triangle curve and logistics curve is used to guide the CV leader to approach and leave the signalized intersection with stable driving behavior when the green light is completely discharged from the queue for planning the speed trajectory of the CV leader.

The main obstacles faced by electric vehicles are the relatively short-range, inadequate infrastructure, and the difficulty of long-distance travel. While the development of batteries and other technologies has enabled future vehicles to overcome these difficulties, it is necessary to reduce energy consumption by adopting more energy-efficient driving behavior. The emergence of advanced sensing technologies and V2X technology opens up new possibilities for security and energy-efficient applications. A model predictive optimization method is proposed [43] where the power system model, traffic light sequence, and other vehicles are used to calculate the energy efficiency speed and shift profile in the limited optimization range, and the phase front and rear dynamic programming method is employed for optimization.

Microscopic eco-speed control algorithms [44] are not like most of the existing research in this area where research focuses on infrastructure control. The authors use the concept of an instrumented vehicles (IV) queue, which connects multiple intersections in a journey and is placed in multiple vehicles, proposing a greedy algorithm based on the driving behavior “stop-and-go” and microscopic fuel and emission models. Seven algorithms are introduced and compared in the simulation and the result comparison is shown in Table 5. The model gives a speed trajectory to reduce fuel consumption and greenhouse gas emissions. The goal is to control the speed of each vehicle at the micro level to reduce fuel consumption and emissions (hydrocarbons and COx). A kind of energy saving variable speed and shift trajectory is obtained by using the forward and backward dynamic programming model predictive optimization method [45]. Since the length of event horizon is limited, a method to create an independent time invariant auxiliary event horizon using the minimum cumulative cost of history is proposed. The auxiliary horizon is used to make a good long-distance estimation of the optimal terminal behavior of the optimal trajectory within the conventional horizon. This method can be applied to different types of optimization problems, but the focus is on predictive energy efficiency optimization of electric vehicles.

#### 3.2.3. Summary of GLOSA

This section focuses on several metrics for evaluating algorithms, including technical and more intuitive criteria, CO${}_{2}$ emission, fuel consumption, and travel time. Then three kinds of algorithms are compared: One is based on ACC, the other is greedy algorithm, and the other is dynamic programming. These algorithms all show a good state for green wave traffic, but few factors, such as road conditions in the actual scene, are not considered. The subsequent algorithms can take the actual interference factors into account to make the results more valuable for reference. We also choose LOSB-F algorithms in [44] to calculate the suitable speed to guarantee vehicles’ passing the intersection without stopping.

### 3.3. Trajectory Planning

#### 3.3.1. Algorithms

**Figure 7:**
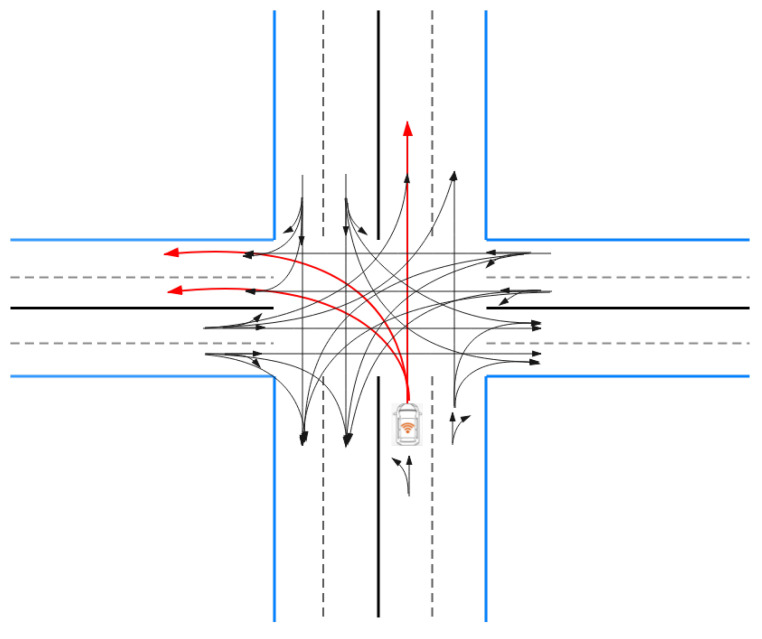
The scene of trajectory planning.

After obtaining the permission of the green wave, using methods of trajectory planning for local planning which is shown in Figure 7, the vehicles pass the intersection. For automated road vehicles, the trajectory planner should meet the requirements of different time scales. First, in just a few seconds, it must guarantee a collision-free trajectory for all road participants and a reasonable safety margin and comfort [47]. The fundamental goal of the trajectory planning work is to manage the driverless vehicle without collision to pass through the intersection, to keep the vehicle running continuously, and to reduce the waiting time of passing the intersection [48]. The trajectory planning methods are mainly divided into two categories: Traditional algorithms and methods based on reinforcement learning.

Traditional algorithms of trajectory planning can be divided into technologies based on graph search and on sampling. The methods of graph search extend the path planning technique to ensure the vehicle state over time. Among the autonomous vehicles, the most commonly used graph search techniques based on trajectory planning techniques are state lattice, elastic-band (EB), and A-star [49]. The state lattice is a search graph in which the vertices represent states, and the edges represent paths that connect states that satisfy the kinematic constraints of the robot. The vertices are placed in the usual way so that the same path can be used to connect all the vertices. This state grid must accommodate road planning by indicating only the states that may be in the solution. In addition, the state lattice must be extended to a dynamic environment by increasing the time and speed dimensions. State grids can handle multiple dimensions, such as position, velocity, and acceleration, and are suitable for local planning and dynamic environments. However, they have high computational costs because it evaluates every possible solution in the diagram. The path planning method based on the elastic band optimization technique [50] uses a graph with elastic nodes and edges to represent the state space. Elastic nodes are defined by adding an inner edge and an outer edge to the spatial node to connect adjacent spatial nodes. A predictive control method [51] based on the model is used to generate the optimal trajectory to avoid collision. In many cases, emergency braking cannot be performed. The safety factor of collision avoidance is implemented in each movement stage of the vehicle, and the nonlinear constraints between the brake and the steering angle are established for emergency motion planning of the vehicle. The efficiency of solution is evaluated through a simulation of different scenarios, which points out the direction for the application research of practical autonomous vehicle problems. The issue of optimal collision planning for vehicle collision avoidance based on the time elastic band (TEB) [52] framework has been studied. The collision avoidance trajectory is given by optimizing the TEB of multiple local optimization targets. The resulting trajectory constitutes the best compromise between simple braking and lane changing, avoiding collisions with the smoothest possible path. Since TEB considers vehicle dynamics, road boundaries, static obstacles, and constraints imposed by moving vehicles, this method is suitable for general critical trajectory situations. An obstacle avoidance trajectory planning method [53] for vehicle sin process can be used to plan the trajectory with continuous curvature and approximately zero longitudinal acceleration in the fixed frame system of a vehicle’s body. Then, according to the characteristics of each subsystem, the trajectory tracking algorithms of the full-drive steering subsystem and the under-drive longitudinal subsystem of the work-in-process vehicle are constructed respectively by using the non-singular terminal sliding mode control and nested saturation method. In the sense of the Lyapunov stability theory, the stability and asymptotic convergence of the tracking error of the closed-loop system are proved. The A-star algorithm is commonly used for path planning or unstructured trajectory planning. Fassbender et al. propose a method named two new A-star [54] that expand the node for road trajectory planning. The first scheme attempts to find a trajectory by numerical optimization, connecting the current node of the vehicle directly to the target node. The second option uses a pure tracking controller to generate the short side (which guides the car along the whole situation reference path). The basic algorithm formula [55] is shown as follows:

(4) f(n)=g(n)+h(n)

During the operation, A* algorithm selects the node with the lowest f(n) value (highest priority) from the priority queue as the node to be traversed next time. N represents node; f(n) is the comprehensive priority of node n; g(n) is the cost of n from the starting point; and h(n) is the expected cost of node n’s distance from the end point, which is also the heuristic function of A* algorithm.

**Figure 8:**
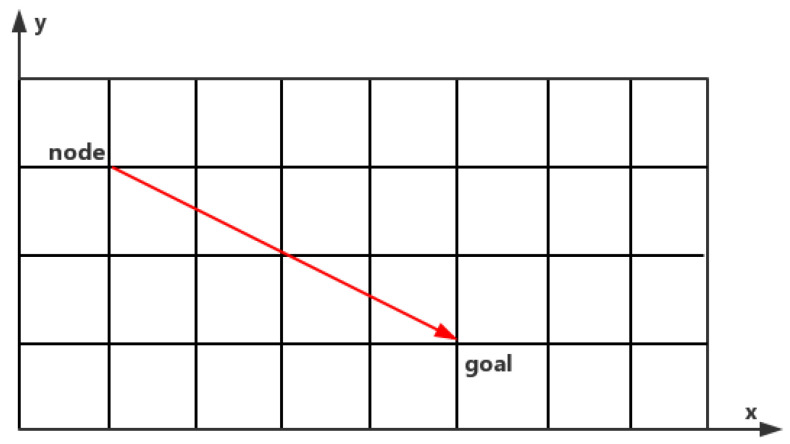
From node to goal.

As for heuristic function h(n), there are three main functions. The first type is Manhattan distance [56]. We need to calculate the distance from node to goal as shown in Figure 8. The calculation is as follows. *D* refers to the movement cost between two adjacent nodes, which is usually a fixed constant.

Function heuristic(node) =

$$d_{x}=abs\left(node.x-goal.x\right)$$

$$d_{y}=abs\left(node.y-goal.y\right).$$

Return (4):

(5) $$D*(d_{x}+d_{y})\;.$$

The second type is diagonal distance [56]. The calculation is as follows. D2 refers to the cost of movement between two obliquely adjacent nodes.

Function heuristic(node) =

$$d_{x}=abs\left(node.x-goal.x\right)$$

$$d_{y}=abs\left(node.y-goal.y\right).$$

Return (5):

(6) $$D*\left(d_{x}+d_{y}\right)+\left(D2-2*D\right)*min\left(d_{x},d_{y}\right).\;$$

The last type is simple and we use it commonly, which names Euclidean distance [56]. The calculation is as follows.

Function heuristic(node) =

$$d_{x}=abs\left(node.x-goal.x\right)$$

$$d_{y}=abs\left(node.y-goal.y\right).$$

Return (6)

(7) $$D*sqrt(d_{x}*d_{x}+d_{y}*d_{y}).\;$$

The technology based on sampling randomly samples the state space to find the connection between the current state of the car and the next target state. In autonomous vehicles, the most commonly used sampling technique based on trajectory planning techniques is a rapidly-exploring random tree (RRT) [57]. The RRT method for trajectory generation uses a random sample in the state space to incrementally construct a search tree from the current state of the car. For each random state, a control command is applied to the nearest vertex of the tree to create a new state that is as close as possible to the random state. Each vertex of the tree represents a state, and each directed edge represents a command for extending the state. Candidate trajectories are evaluated according to various criteria. The RRT method based on sampling has a lower computational cost for high-dimensional space, and as long as there is a solution and time is sufficient, the solution can always exit. However, the results are not continuous and stable. A rough trajectory is obtained by using a sampling search planner, and then it is optimized by numerical optimization. Among the mainstream sampling search planners, most sampling operations are not flexible. If the sampling density is low, the solution will inevitably fail; if the sampling density is high, the dimension curse will appear. In this paper, authors propose an improved RRT* algorithm [58] for trajectory search, which uses a sampling search planner to get a rough trajectory, and then optimizes it through numerical optimization, so as to improve the sampling flexibility of trajectory search and eliminate the randomness of search. A new smoother based on quadratic programming (QP) is proposed to refine coarse trajectories.

**Figure 9:**
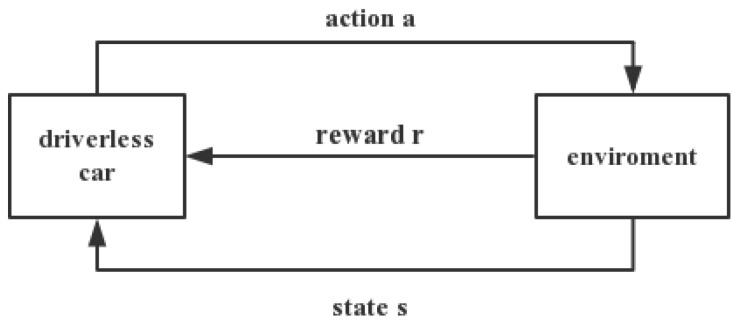
The frame of reinforcement learning.

Reinforcement learning (RL) usually include two parts: Agent and environment. The frame is shown in Figure 9. The interaction of two parts is as follows: The agent, which is under the environment of state $s_{t}$, chooses action to get the reward $r_{t}$ and enter into the state $s_{t+1}$. RL in the field of driverless means that unmanned vehicles use their own sensors to constantly interact with the environment to acquire knowledge of the unknown environment. RL has the advantage of learning online through trial and error through interaction with the environment, acquiring knowledge in the environment of action and evaluation, and improving action plan [59]. The paper introduces three methods: Instantaneous difference method, Sarsa method, and Q-learning which is the more effective environment-model-independent algorithm and can be learned online.

**Figure 10:**
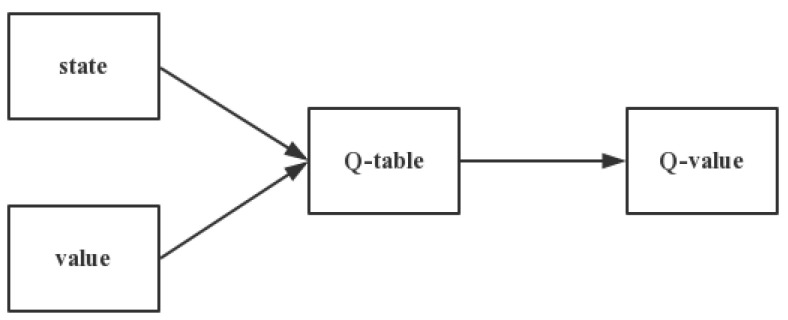
Process of Q-learning.

The Q-table, whose row and column respectively represent the value of state and action is the core of Q-learning, is shown in Figure 10. Thus, the value Q(s,a) of Q-table is used to measure whether the behavior that state ‘s’ adopt action ‘a’ is fine [60].

In the process of training, the algorithm uses the Bellman Equation to update the Q-table. The formula is as follows [61]:

(8) $$Q\left(s,a\right)=r+\gamma *max[Q\left(s^{\prime },a^{\prime }\right)].\;$$

Although RL is efficient in trajectory planning under stochastic dynamic environment, it is also a challenge and a research problem in speeding up the convergence of the algorithm, reducing space complexity, and improving learning ability in the environment. Reinforcement learning based on neural network can improve the problem of insufficient storage space, approaching the Q function through the neural network, and constantly updating the value of Q after obtaining the state of unmanned vehicle. The neural networks is trained according to the BP algorithm and the path planning is completed.

In addition to the above algorithms, various intelligent information optimization methods have also been introduced into the field of path planning. The intelligent water drop algorithm [62], ant colony algorithm [63], and tentacle algorithm [64] all improve the algorithm performance to a certain extent and enhance the quality of trajectory planning.

Table 6 compares the algorithms from three aspects: Real-time performance, robustness, and time complexity. It can be seen that the RRT algorithm in the traditional algorithm has better performance in real-time and robustness, and the A* search rate is faster; the algorithm based on reinforcement learning has a certain improvement in real-time and robustness compared to the traditional algorithm, but the time complexity is worse.

#### 3.3.2. Summary of Trajectory Planning

This part mainly introduces several common algorithms of trajectory planning and compares their performance. At present, A* algorithm and Q-learning based planning method are more commonly used. Relatively speaking, the performance of Q-learning is improved, so we can focus on optimizing the relevant algorithms based on Q-learning in future research.

### 3.4. Emergency Vehicle Priority

**Figure 11:**
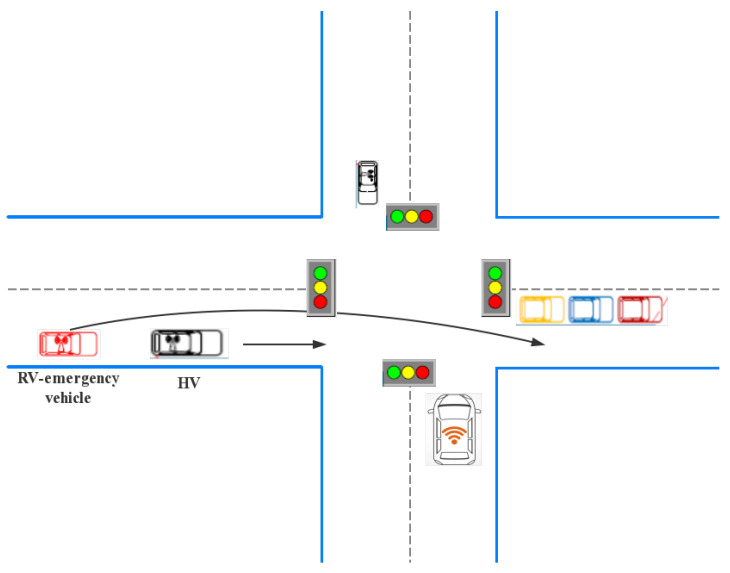
Priority for emergency vehicle scheduling.

This section proposes a particular vehicle: The emergency vehicle. Statistics show that emergency vehicles are more prone to accidents than other road users when performing tasks, especially at intersections, as is shown in Figure 11. Statistics on the German rescue service mission show that there are more than 14 million missions per year, which is equivalent to having tens of thousands of emergency vehicles perform tasks every day. Each task is carried out under tremendous time pressure because the local response time controls the maximum time difference between the incoming call and the arrival of the rescue team [65]. The emergency vehicle preemption system allows emergency vehicles such as fire fighting trucks and ambulances to request and receive green traffic signal indications as they approach an intersection [66]. Emergency Vehicle Preemption (EVP) provides a green belt along the route at isolated and coordinated signal intersections, interrupting normal traffic signal timing operations. EVP allows the EVs to pass without stopping or waiting at the intersection, which may reduce travel time and reduce conflict with other vehicles in the system.

Ma et al. [67] study the priority of EV signals from different directions and necessarily pass the same intersection in a specific period time, and propose a multi-agent EV signal priority control system based on multi-agent. The phase agent and management agent are imported in the system, and the fuzzy logic theory is used to realize the internal logic of various agents and the coordination mechanism between modules. The Dynamic Preemption Algorithm (DPA) [68] is based on the idea of adjusting the traffic signal cycle at each intersection along the road to ensure sufficient green light to allow the emergency vehicles to pass through the intersection with minimal delay without stopping. The algorithm must determine the emergency vehicle at a particular location and then decide the exact seconds at which the preemption must begin in the traffic signal phase period based on the Global Positioning System (GPS) information received from the EV. The decision of position must strike a balance between two essential requirements: One is that the minimum travel time for an emergency car should approximately equal to its theoretical free travel time, the other is that the additional delay for other vehicles should reach the minimum or be slight enough at the same intersection.

The algorithms comparison of the aboved are shown in Table 7. Many of the developed preemption strategies are based on a single intersection. These strategies rely on local detection and clearing intersections one by one, and the signal preemption process cannot be initiated before an emergency vehicle is detected; the inherent delays at the intersection are unavoidable. This signal preemption strategy can only be initiated after the detection of the emergency vehicles, which often leads to inevitable intersection delay. Mu et al. propose a signal preemption control method named Timed Colored Petri Nets (TCPN) to reduce the delay of emergency vehicles at intersections [69]. TCPN is used to establish a traffic flow model, traffic signal display, phase switching model, and traffic signal switching control model. An urban transportation network model consists of three sub-models. An emergency vehicle preemptive optimization control system is designed, including monitoring subsystem, phase time determination subsystem, and phase switching control subsystem. Louati et al. propose a method [70] that facilitates the passage of emergency vehicles through urban intersections. They rely on preemptive technology and multi-agent systems. Benchmarking and analysis are performed using VISSIM traffic simulation software. The article considers several metrics to assess network performance, including latency, travel time, vehicle queue occupancy, number of stops, distance travelled, and speed.

## 4. Conclusions

This paper focused on the safety of intersection and efficiency improvement by the fast development of vehicle-to-vehicle and vehicle-to-infrastructure communications. The overview of methods for intersection management is also presented and all of these are useful to improve the traffic condition. A relatively good method was chosen from each scene to construct a solution that was useful to pass through the intersection.

In future, virtual traffic lights will more likely become an attractive and mutual way to ease traffic pressure. Intelligent collaboration between connected vehicles and roadside units provides new opportunities for better road safety and vehicle traffic management via innovative non-signalized intersections. We could build the road-network-wide conflict-free geometric topology considering the vehicles’ conflicting relationships. To deal with sophisticated traffic information brought about by a dynamic non-signalized intersection environment, artificial intelligence (AI) solutions together with V2X communication technologies are proposed to provide data-driven intersection management strategies. AI technologies that have been applied for vehicle traffic scheduling include reinforcement learning, artificial neural networks, and mutil-agent systems. Algorithms in these three directions can be employed separately or jointly. Intersection manager and vehicles are normally looked upon as intelligent agents. Via a virtual traffic model projected in the real world or directly using a practical traffic environment, agents are capable of obtaining an optimal scheduling strategy based on the received feedback under different situations. Non-signalized intersection management mechanisms can potentially leverage various AI technologies to achieve road safety and enhance traffic management efficiency.

## Figures and Tables

**Table 1:** Performance criteria of test scenes.

Criteria	Data Range
HV speed	0–130 km/h
Communication distance	>= 300 m
Position accuracy	<= 5 m

**Table 3:** Three types of abnormal information warning.

Type	Usage Scenario	Zone of Application	Examples
Abnormal vehicle warning	Radar, laser, lidar, etc.	Intersections; roads of cities and suburban	IVMS [7]
Control loss warning	ABS, ESP, TCS, and LDW of RV	Roads of cities and suburban	IDAS [14]
Hazardous location warning	Potentially dangerous condition (e.g., holes of road surface)	Road bends; the bad situation of road	Interchange server [15]

**Table 4:** Summary of methods in congestion avoidance.

Type	Model	Main Technology	Advantages	Need To Be Improved
Using prediction algorithm	AUTM [31]	Map and transfer; optimized spatial evolution rules; a congestion avoidance routing algorithm	Congesting forecasting more than 89%	Considering more realistic features in AUTM; applying dynamic strategy of traffic light; short of real-time
	An advanced dynamic traffic network prediction and navigation model [32]	Equilibrium Markov chain model; time-dependent navigation algorithm	Average speed improved	Network adaptation and robustness; making the network into real-time; self-update
VANET	A real-time queue length estimation method [35]	Using Markov model to estimate the real-time CV; the real-time queue length estimation	Good performance in handling the randomness; high accuracy in real-time queue length estimation	The impact of queue estimation accuracy caused by different CV distributions in the queue

**Table 5:** Performance comparison of GLOSA.

Algorithm	CO${}_{2}$ Emission Reduction	Fuel Consumption Reduction	Travel Time Reduction
An extension of IDM and eco-driving model [41]	7.9%	2.3%	1.5%
A model predictive optimization approach [43]	6.2%	9%	4.1%
GDA [46]	6.8%	7.1%	6.8%
MCG [44]	5.7%	4.2%	3.4%
MCG-EA [44]	6.1%	2.1%	2.4%
LOSB-F [44]	6.3%	1.5%	2.0%
LOSB-E [44]	6.4%	2.5%	1.7%

**Table 6:** Algorithms performance of trajectory planning.

Algorithm	Real-Time	Robustness	Time Complexity
State lattice [49]	85%	++	++++
EB [50]	75%	+	++++
A* [54]	88%	++	+++
RRT [57]	79%	++	++
Q-learning [60]	90%	+++++	++

**Table 7:** Algorithms comparison of emergency vehicle priority.

Algorithm	Method	Result
The multi-agent EV signal priority control system [67]	Fuzzy logic theory; phase agent and management agent	Passing time decreases by 16.4% and stop times reduce by 8.21%
DPA [68]	The acyclic signal operation based on a real time control approach; Passing Vehicle Search algorithm (PVS)	The delay decreases by 12.51%
TCPN [69]	Establish a traffic flow model; traffic signal display and phase switching model and traffic signal switching control model	The maximal improvement rate compared with the traditional method is 29.49%
A distributed Traffic Signal Control System (TSCS) [70]	LQF-MWM assumptions, preemption technique; multi-agent system	The system reduces the total travel time by 11.7% and 27.02% compared to MAS-P-OSBFX-240S and MAS-P-OSBFX-60S

## Data Availability

The data presented in this study are available on request from the corresponding author. The data are not publicly available due to privacy.

## Abbreviations

The following abbreviations are used in this manuscript:

anti-lock braking system	ABS
Adaptive Cruise Control	ACC
Abnormal Vehicle Warning	AVW
body stability system	BSS
Control Loss Warning	CLW
convolutional neural network	CNN
connected vehicles	CVs
Dynamic Preemption Algorithm	DPA
elastic-band	EB
Emergency Vehicle Preemption	EVP
green light optimized speed advisory	GLOSA
Global Positioning System	GPS
Hazardous Location Warning	HLW
Hopfield Neural Network	HNN
host vehicle	hv
hybrid wavelet decomposition	HWD
Intelligent Driver Assistance System	IDAS
intelligent prediction model	IPM
intelligent traffic system	ITS
instrumented vehicles	IV
in-vehicle monitoring system	IVMS
lane offset warning system	LOW
map and transfer	MT
radio frequency identification	RFID
Reinforcement learning	RL
rapidly-exploring random tree	RRT
roadside units	RSU
remote vehicle	RV
signal phase and time	SPaT
support vector machines	SVMs
Timed coloured Petri nets	TCPN
traction control system	TCS
time elastic band	TEB
Traffic Signal Control System	TSCS
Vehicle-To-Infrastructure	V2I
Vehicle-To-Network	V2N
Vehicle-To-Pedestrian	V2P
Vehicle-To-Vehicle	V2V
Vehicular Adhoc Networks	VANETs
signal phase and time	SPaT
wavelet zero padding	WZP
artificial intelligence	AI
